# Transcriptional control of embryonic and adult neural progenitor activity

**DOI:** 10.3389/fnins.2023.1217596

**Published:** 2023-07-28

**Authors:** Niharika Singh, Florian A. Siebzehnrubl, Isabel Martinez-Garay

**Affiliations:** ^1^Division of Neuroscience, School of Biosciences, Cardiff University, Cardiff, United Kingdom; ^2^European Cancer Stem Cell Research Institute, Cardiff University School of Biosciences, Cardiff, United Kingdom

**Keywords:** neurogenesis, transcription factor, embryonic, adult, bHLH, homeodomain, forkhead, Zeb1

## Abstract

Neural precursors generate neurons in the embryonic brain and in restricted niches of the adult brain in a process called neurogenesis. The precise control of cell proliferation and differentiation in time and space required for neurogenesis depends on sophisticated orchestration of gene transcription in neural precursor cells. Much progress has been made in understanding the transcriptional regulation of neurogenesis, which relies on dose- and context-dependent expression of specific transcription factors that regulate the maintenance and proliferation of neural progenitors, followed by their differentiation into lineage-specified cells. Here, we review some of the most widely studied neurogenic transcription factors in the embryonic cortex and neurogenic niches in the adult brain. We compare functions of these transcription factors in embryonic and adult neurogenesis, highlighting biochemical, developmental, and cell biological properties. Our goal is to present an overview of transcriptional regulation underlying neurogenesis in the developing cerebral cortex and in the adult brain.

## Introduction

Neurogenesis happens primarily during embryonic stages, while the nervous system develops, although some regions in the adult brain retain the capacity to generate new neurons throughout life (Jurkowski et al., 2020). In both cases, neural progenitors need to balance their own proliferation with the production of differentiated cells to ensure that appropriate numbers of neurons and glia are made. During embryonic neurogenesis, progenitors first proliferate through symmetric divisions until about E11.5, when they change their division mode and start producing neurons through asymmetric divisions. Once all necessary neurons have been generated, they will begin generating glial cells in an irreversible switch that signifies the end of embryonic neurogenesis (Martynoga et al., 2012). Because transitions between phases cannot be reversed, accurate control of proliferation vs. differentiation is paramount to ensure the correct development of the nervous system. Postnatally, some radial glial cells become the specialized neural stem cells (NSCs) for postnatal and adult neurogenesis (Bond et al., 2020). In neurogenic regions [subventricular zone, hippocampus and hypothalamus, reviewed in Jurkowski et al., 2020], NSCs generate intermediate progenitor cells through asymmetric division. Intermediate progenitor cells expand rapidly and eventually differentiate into neuronal progenitor cells that migrate to their destination where they integrate into neuronal circuitry upon terminal differentiation. Contrastingly to embryonic neurogenesis, adult NSCs can simultaneously generate glial cells through a much less understood process.

The balance between proliferation and differentiation of neural stem and progenitor cells requires exquisite control at the transcriptional level. Transcriptional control of embryonic and adult neurogenesis relies on shared transcription factors (TFs) that become spatially confined over time, are expressed at specific timepoints, or both. The cell type-specific transcriptional activity of such neurogenic TFs is mediated by epigenetic signatures, chromatin changes, and other protein partners. In this mini-review, we provide a brief overview focusing on the role of some of the best-characterized TFs that control neurogenesis in the embryonic dorsal telencephalon (Table 1) and in the adult subgranular zone (SGZ) of the hippocampus and the subventricular zone (SVZ) of the lateral ventricles (Table 2). For more comprehensive analyses of the role of specific TFs, we refer the reader to appropriate reviews.

**Table 1:** Role of different transcription factors in embryonic neurogenesis.

DNA-binding domain	Transcription factor	Role	References
Basic helix–loop–helix (bHLH)	HES1	Represses neuronal differentiation of NSC pool	Ishibashi et al. (1994), Nakamura et al. (2000), Shimojo et al. (2008), Dhanesh et al. (2016), and Gozlan and Sprinzak (2023)
		Heterogenous differentiation of NSCs	Kobayashi et al. (2009) and Kobayashi and Kageyama (2011)
	NGN2	Proneural differentiation of NSCs, regulation of progenitor maturation and of neuronal vs. glial fate decision	Nieto et al. (2001), Parras et al. (2002), Britz et al. (2006), Mattar et al. (2008), Miskinyte et al. (2018), and Han et al. (2021)
	ASCL1	Lineage commitment of NPCs to neuronal fate	Castro et al. (2011) and Vasconcelos and Castro (2014)
		Defines neurogenic patterning and cortical folding	Han et al. (2021)
Homeodomain	PAX6	Controls balance between neural stem cell (NSC) self-renewal and neurogenesis	Estivill-Torrus et al. (2002), Sansom et al. (2009), Mi et al. (2013), and Manuel et al. (2015)
		Dorsoventral patterning of the mammalian telencephalon	Toresson et al. (2000) and Yun et al. (2001)
	SOX2	Promotes progenitor proliferation and prevents differentiation	Miyagi et al., 2008
Zinc finger + leucine zipper + forkhead domain	FOXG1	Maintains balance between proliferation and differentiation in neural progenitors	Xuan et al. (1995), Dou et al. (1999), Hanashima et al. (2002), Shen et al. (2006), Eagleson et al. (2007), and Siegenthaler et al. (2008)
	FOXM1	Maintains stem cell pluripotency and self-renewal capacity of stem cells	Kalin et al. (2011) and Wu et al. (2014)
	FOXP1	Maintains progenitor pool by promoting progenitor self-renewal	Pearson et al. (2020)
		Promotes progenitor differentiation	Braccioli et al. (2017)
	FOXP2	Induces generation of intermediate progenitors	Tsui et al. (2013)
	FOXP4	Promotes progenitor differentiation	Rousso et al. (2012) and Li et al. (2023)
	FOXO 1/3/4	Mediate antiproliferative TGF-B signaling in early neural progenitors	Seoane et al. (2004)
Zinc finger homeodomain	ZEB1	Neuronal differentiation, and migration	Jiang et al. (2018) and Wang et al. (2019)
		Cleavage plane orientation in progenitors	Liu et al. (2019)

**Table 2:** Role of different transcription factors in postnatal/adult neurogenesis.

DNA-binding domain	Transcription factor	Role	References
Basic helix–loop–helix (bHLH)	HES1	Promotes/ regulates quiescence and proliferation of NSCs	Zhang et al. (2015), Sueda et al. (2019), and Kaise and Kageyama (2021)
	NGN2	Neuronal differentiation of progenitors	Ozen et al. (2007), Roybon et al. (2009), and Arai et al. (2017)
	ASCL1	Activation of quiescent NSCs, drives differentiation of NSPCs to neurogenic fate	Imayoshi et al. (2013), Andersen et al. (2014), Urbán et al. (2016), Pilz et al. (2018), and Harris et al. (2021)
		Defines SGZ and SVZ cells with long-term neurogenic potential	Kim et al. (2011)
Homeodomain	PAX6	Generation of neuronal progenitors and their specification into dopaminergic periglomerular phenotype	Hack et al. (2005), Kohwi et al. (2005), and Brill et al. (2008)
	SOX2	NSC maintenance	Ferri et al. (2004) and Favaro et al. (2009)
Zinc finger + leucine zipper + forkhead domain	FOXG1	Proliferation of neuronal progenitors in neurogenic niches	Shen et al. (2006), Tian et al. (2012), and Wang et al. (2022)
	FOXJ1	Maintains progenitor proliferation in SVZ through cell autonomous and non-autonomous mechanisms	Jacquet et al. (2009) and Jacquet et al. (2011)
	FOXO 1/3/4	Maintains the population of quiescent NSCs	Renault et al. (2009), Webb et al. (2013), Li et al. (2017), and Schäffner et al. (2018)
Zinc finger homeodomain	ZEB1	Self-renewal of active radial glia-like cells to favor an astroglial fate, shift in cell division polarity	Gupta et al. (2021)

## bHLH transcription factors in neurogenesis

Transcription factors of the bHLH (basic helix loop helix) superfamily work as dimers and bind DNA through a basic domain at their amino terminal end (Jones, 2004). Several bHLH TFs play important and sometimes opposing roles during embryonic and adult neurogenesis.

HES (Hairy and Enhancer of Split homologs) family members of the bHLH TF family are effectors of the Notch signaling pathway (Ohtsuka et al., 1999). During corticogenesis, they regulate cell proliferation, differentiation, and fate specification by maintaining stemness of progenitors and controlling the timing of differentiation (Kageyama et al., 2007; Gozlan and Sprinzak, 2023) in neuroepithelial and radial glial cells. Of the different *Hes* genes, *Hes1* is the most widely studied in the context of corticogenesis. HES1 levels in cortical neuronal progenitors experience cyclic oscillations, which are essential for the maintenance of neuronal progenitors (Shimojo et al., 2008). These oscillations result from the combination of *Hes1* expression induction by Notch signaling, an autoinhibitory effect of HES1 on its own transcription and the great instability of the *Hes1* mRNA and protein (Takebayashi et al., 1994; Hirata et al., 2002). In its capacity as an antineurogenic bHLH repressor (Nakamura et al., 2000), HES1 acts in two different ways. First, it represses expression of its target genes by directly binding to their promotors in a complex with co-repressors like Groucho/TLE-1 (Jiménez et al., 1997; Dhanesh et al., 2016). Second, HES1 interferes with the transcriptional activity of target TFs by binding to and sequestering E proteins such as E47, which are required by TFs like ASCL1 to function (Sasai et al., 1992; Dhanesh et al., 2016). Downstream targets of HES1 include cell-cycle regulators like the CDK inhibitor *Cdkn1B* (Murata et al., 2005), *Gadd45g*, cyclins D2 and E2, and the Notch ligand *Dll1* (Shimojo et al., 2008). In addition, HES1 also inhibits expression of several proneural bHLH TFs, including *Ascl1* and *Neurog2* (Shimojo et al., 2008). HES1 fluctuations drive oscillatory expression of these TFs and help maintain the progenitor population, especially during early stages of corticogenesis (Shimojo et al., 2011). In turn, expression, or lack thereof of the proneural bHLH TFs *Ascl1* and *Neurog2* define four different progenitor states, with expression of both TFs representing the least lineage restricted progenitors and those expressing only *Neurog2* committed to a neuronal lineage (Han et al., 2021). Furthermore, combined expression of *Ascl1* and *Neurog2* leads to cross-repression and to the production of Notch ligands that maintain proliferation in neighboring cells (Han et al., 2021).

During adult neurogenesis, sustained levels of HES1 are needed to keep aNSCs in the SVZ and SGZ in a quiescent state (Sueda et al., 2019), as constant, high HES1 indirectly leads to increased CDKN1A levels, inhibiting cell cycle progression (Maeda et al., 2023). This is accomplished through the interaction of HES1 with ID1, which represses HES1 autoinhibition (Bai et al., 2007). As NSCs activate, oscillating expression of HES1 drives a concomitant oscillatory expression of ASCL1, which is critical for NSC activation (Andersen et al., 2014). In fact, lower levels of ASCL1 are linked to higher numbers of resting NSCs (Urbán et al., 2016) and a proliferation vs. differentiation bias in progenitors (Imayoshi et al., 2013), while ASCL1 protein levels drop over time to ensure the maintenance of the aNSC pool (Harris et al., 2021).

## Homeobox transcription factors in neurogenesis

There are 11 different classes of homeobox transcription factors, characterized by a helix-turn-helix homeodomain motif that mediates their binding to DNA (Holland et al., 2007). We discuss PAX6 and SOX2 here, but the roles of 21 homeobox TFs in vertebrate forebrain development have been comprehensively reviewed elsewhere (Leung et al., 2022).

PAX6 belongs to the paired-box homeodomain transcription factor family, harboring a second DNA binding domain, the paired box, in addition to the homeodomain (Dahl et al., 1997). PAX6 is one of the main regulators of cortical neurogenesis, controlling cell cycle length and exit in a dose and context dependent manner [reviewed in Manuel et al. (2015)]. As such, loss of *Pax6* leads to shorter cell cycle length and a premature switch from proliferative to neurogenic divisions during early neurogenesis, with more pronounced effects in areas of higher *Pax6* expression (Estivill-Torrus et al., 2002; Mi et al., 2013). However, at later stages, *Pax6* loss leads to a longer cell cycle (Estivill-Torrus et al., 2002) and its overexpression decreases the number of proliferating progenitors in rostral and medial areas at E15.5 (Manuel et al., 2006). These results highlight the context dependent actions of this TF, which is needed both for progenitor proliferation and for neurogenesis. Interestingly, the effects of PAX6 during corticogenesis, except for its patterning role, are mediated by the paired-box, and not by the homeodomain (Haubst et al., 2004). PAX6 regulates progenitor cell proliferation in part by controlling expression of several genes involved in the G1/S transition, including cyclins and *Cdks* (Sansom et al., 2009; Mi et al., 2013). PAX6 has been shown to directly inhibit *Cdk6* expression, thereby reducing Rb phosphorylation and slowing down G1 progression (Mi et al., 2013). Regarding neurogenesis, PAX6 directly induces expression of *Tbr2*, which confers intermediate progenitor identity (Quinn et al., 2007; Sansom et al., 2009). In addition, PAX6 also stimulates expression of *Neurog2*, and participates in a transcriptional network with NEUROG2, ASCL1 and HES1 to control the outcome of neural progenitor cell division (Sansom et al., 2009).

During adult neurogenesis, PAX6 seems to play a similar role controlling proliferation and neuronal differentiation of aNSCs (Hack et al., 2005; Maekawa et al., 2005). In the SGZ, PAX6 induces expression of *Neurog2* and *NeuroD1* (Xu et al., 2021), which are needed to maintain NSC progenitors and induce neuronal fate, respectively (Roybon et al., 2009). It also acts through FABP7 to maintain NSC and progenitor cell proliferation and prevent exhaustion of the stem cell pool (Osumi et al., 2008). In the SVZ and the rostral migratory stream (RMS), PAX6 is needed to regulate neuronal precursor proliferation and for periglomerular neuron fate (Hack et al., 2005).

SOX2 (SRY-box binding transcription factor 2) is a member of the Sox family of transcription factors, which consists of 9 subfamilies (*SoxA, SoxB1, SoxB2, SoxC, SoxD, SoxE, SoxF, SoxG, SoxH*). *Sox2* is part of the *SoxB1* subgroup (together with *Sox1* and *Sox3*; Wegner, 2010) and is a master regulator of stemness in development and adult tissues (Sarkar and Hochedlinger, 2013). It is a pioneer factor that can initiate transcription in epigenetically silenced chromatin regions (Dodonova et al., 2020). SOX transcription factors bind the consensus sequence TTGT through their high-mobility-group (HMG) box (Wegner, 2010), with specificity of individual SOX factors conveyed by DNA regions flanking the consensus motif (Sarkar and Hochedlinger, 2013). SOX2 is expressed throughout embryonic and adult neurogenesis, as well as in pluripotent embryonic stem cells and *Sox2* knockout (KO) is lethal during early embryogenesis (Avilion et al., 2003). In the developing brain SOX2 promotes progenitor proliferation and prevents cell differentiation, functions that overlap with SOX1 and SOX3 (Wegner and Stolt, 2005; Miyagi et al., 2008). Interestingly, Sox2 hypomorphism also affects differentiation into GABAergic interneurons in the cortex and olfactory bulb at E17.5 (Cavallaro et al., 2008).

In the adult CNS, SOX2 is expressed in all neurogenic niches, and conditional deletion of *Sox2* results in impaired NSC proliferation, increased apoptosis, and reduced neurogenesis in the SVZ and SGZ (Ferri et al., 2004; Favaro et al., 2009). The wide-ranging functions of SOX2 in the brain are reviewed in more detail in Pevny and Nicolis (2010) and Mercurio et al. (2019).

## Forkhead transcription factors in neurogenesis

Forkhead transcription factors are characterized by the presence of the so-called forkhead domain, which mediates their interaction with DNA. This domain consists of three α-helices and three β-sheets surrounded by two loops that form the “winged” region (Hannenhalli and Kaestner, 2009). Forkhead family members are classified into 19 subfamilies from *FoxA* to *FoxS* (Jackson et al., 2010). Members of the *FoxG, FoxJ, FoxM, FoxO*, and *FoxP* subfamilies have been implicated in embryonic and/or adult neurogenesis and play sometimes opposing roles in the regulation of neural stem cell behavior.

*Foxg1* KO animals die at birth with severe brain hypoplasia (Xuan et al., 1995; Dou et al., 1999) and heterozygous animals display decreased cortical, hippocampal and striatal size, along with reduced numbers of TBR2+ intermediate progenitors (Shen et al., 2006; Eagleson et al., 2007; Siegenthaler et al., 2008). Those changes reflect the role of FOXG1 in maintaining the correct balance between proliferation and differentiation in neural progenitors, with lack of *Foxg1* leading to lengthening of the cell cycle and premature cell cycle exit (Xuan et al., 1995; Hanashima et al., 2002). At the molecular level, FOXG1 antagonizes TGF-B signaling by repressing the expression of TGF-B family members BMP2, 4, 6, and 7, which are all ectopically upregulated in *Foxg1* KOs. This repression requires the DNA binding domain of FOXG1 (Dou et al., 1999; Hanashima et al., 2002). FOXG1 also interferes with the ability of the TGF-B signaling effectors SMADs to promote expression of CDK inhibitors. The SMAD partner FAST-2 is needed for the transcriptional activation of *Cdkn2b*, but binding of FOXG1 to FAST-2 interferes with TGF-B signaling and antagonizes its growth inhibition effects (Dou et al., 2000). To activate *Cdkn1a* expression, SMAD proteins need to form a complex with members of the FOXO subfamily (Seoane et al., 2004). FOXG1 can reduce *Cdkn1a* expression levels by repressing expression of *Foxo1* (Vezzali et al., 2016). Furthermore, FOXG1 interacts with FOXO at the protein level, forming a ternary complex with SMADs that can no longer activate *Cdkn1a* expression (Seoane et al., 2004). In addition, FOXG1 inhibition of *Cdkn1a* expression can also be mediated by its interaction with the polycomb protein BMI-1 (Fasano et al., 2009). FOXG1 could also potentially interfere with the expression of *Cdkn1b*, as its expression is stimulated by BMP treatment (Nakamura et al., 2003; Sharov et al., 2006) and by expression of *Foxo1*, 3 and 4 (Medema et al., 2000).

FOXG1 has also been indirectly linked to Notch signaling, as it interacts with TLE1, which enhances the repressive ability of FOXG1 (Yao et al., 2001). This interaction has been shown *in vitro* and in the E15.5 developing telencephalon. Moreover, TLE1 enables the interaction between FOXG1 and HES1, which increases HES1-mediated transcriptional repression (Yao et al., 2001), suggesting that FOXG1 might act to amplify the effect of Notch signaling in early neural progenitors, as all three proteins are expressed in cultures derived from E12.5 telencephalic progenitors.

Other forkhead family members are also involved in neurogenesis. FOXM1 stimulates expression of genes needed for G1/S transition and DNA replication, while simultaneously diminishing protein stability of CDK inhibitors (Kalin et al., 2011). These roles could explain why cortical progenitors derived from E14 ER^T2^Cre *FoxM1*^fl/fl^ animals display a reduction in the number of neurospheres formed after tamoxifen addition (Wang et al., 2011). FOXM1 also regulates expression of *Sox2* and Bmi1, which are necessary for neural progenitor self-renewal (Wang et al., 2011). However, conditional deletion of *Foxm1* does not lead to major abnormalities in the brain (Schüller et al., 2007), suggesting the presence of compensatory mechanisms. From the FOXP family members, FOXP1 works to maintain the progenitor pool by promoting progenitor self-renewal, at least in part through the induction of vertical division angles and symmetric divisions (Pearson et al., 2020). However, FOXP1 has also been shown to inhibit Notch signaling in the developing cortex, thereby promoting progenitor differentiation (Braccioli et al., 2017). FOXP2 might regulate the generation of TBR2+ intermediate progenitors (Tsui et al., 2013) and FOXP4 promotes neuronal differentiation of neural progenitors by repressing N-Cadherin expression, therefore favoring detachment from the ventricular zone (Rousso et al., 2012; Li et al., 2023).

FOXG1 is strongly expressed in the SGZ of the dentate gyrus and the lateral ventricle SVZ (Shen et al., 2006; Schäffner et al., 2023). In aNSCs of the DG, FOXG1 plays a similar role of balancing proliferation and differentiation as it does in embryonic progenitors (Wang et al., 2022), with partial or total loss leading to defects in size and morphology of this anatomical structure. Progressive loss of progenitors, altered neuronal differentiation and reduced neuronal survival have been described in these mutant animals, as well as a failure to form the secondary radial glia scaffold (Shen et al., 2006; Tian et al., 2012). Remarkably, generation of olfactory interneurons in the SVZ does not seem to be affected by heterozygous lack of *Foxg1* (Shen et al., 2006), suggesting a region-specific function of FOXG1 in adult neurogenesis.

FOXO1 and FOXO3 are also expressed in aNSCs of the SVZ and the SGZ (Paik et al., 2009; Renault et al., 2009). In a *Foxo1/3/4* triple mutant, aNSCs get depleted over time due to decreased self-renewal and increased activation of progenitors early on (Paik et al., 2009; Schäffner et al., 2018). These effects are due to increased expression of cyclins and CDKs and decreased expression of CDK inhibitors, as well as derepression of the centrosomal gene *Aspm*, a known regulator of NSCs divisions (Paik et al., 2009). Very similar results are obtained in *Foxo3* single KO animals (Renault et al., 2009). Transcriptional analysis has revealed that FOXO3 targets are enriched in cell quiescence-related genes, oxidative stress response and cell metabolism, further supporting the notion that FOXO proteins are necessary to maintain the population of quiescent NSCs over the lifespan of the animals by preventing excessive cell cycle reentry (Renault et al., 2009; Ro et al., 2013). This transcriptional control is mediated in part by the interaction of FOXO3 with the methylcytosine dioxygenase TET2 (Li et al., 2017). Moreover, FOX3 restricts the neurogenic effects of ASCL1 in adult NPCs by preventing ASCL1-dependent transcription (Webb et al., 2013) Additionally, lack of *Foxo3* also impacts the outcome of aNSC progeny, with a bias toward astrocytes and reduced production of neurons and oligodendrocytes (Renault et al., 2009).

Finally, FOXJ1 has also been linked to adult neurogenesis in the SVZ of the lateral ventricle. This TF is required for ependymal cell specification during the transition to postnatal stages (Jacquet et al., 2009), but it also defines a subpopulation of progenitors that rely on FOXJ1 expression for its proliferative ability (Jacquet et al., 2011). FOXJ1 deficient progenitors produce fewer neurospheres and are biased toward a glial fate, with defective neurogenic potential. Interestingly, beyond the cell autonomous effect of FOXJ1 in the FOXJ1+ lineage, an additional non-autonomous effect on the remaining aNSCs in the SEZ has been described (Jacquet et al., 2011).

## ZEB1 in neurogenesis

The transcription factor ZEB1 (zinc finger E-box binding homeobox 1) is emerging as a new regulator of self-renewal and fate choice in the CNS. The ZEB family of TFs consists of two members, *Zeb1* and *Zeb2*, which are both core regulators of epithelial-mesenchymal transition (Vandewalle et al., 2009). Epithelial-mesenchymal transition is developmental program that has more recently garnered attention for its role in stemness and lineage regulation (Goossens et al., 2017). *Zeb1*-mutant mice show aberrant T cell development, underlining its involvement in lineage regulation (Higashi et al., 1997). Structurally, ZEB proteins comprise of two C_2_H_2_-type zinc finger domains that flank a central homeodomain. The zinc finger domains are necessary for DNA binding, with each zinc finger independently binding to separate E-box motifs in gene promoters with the consensus sequence 5′-CACCT(G)-3; Sekido et al., 1996, 1997; Remacle et al., 1999). The homeodomain mediates interaction with other proteins (e.g., CTBP, YAP) that are necessary for transcriptional regulation (Furusawa et al., 1999; Feldker et al., 2020). Depending on their interaction partners, ZEB TFs can activate or repress transcription, with E-cadherin repression and Vimentin activation being the best-known examples (Vandewalle et al., 2009). Phosphorylation of Thr-867 is necessary for nuclear import of ZEB1 (Llorens et al., 2016), otherwise the effects of post-transcriptional ZEB1 modifications are poorly understood.

In embryonal neurogenesis, ZEB1 is expressed in the subventricular zone and overlaps with proliferating progenitor cells between E14 and E18 (Yen et al., 2001). Constitutive deletion of *Zeb1* causes defects in proliferation of embryonic neural progenitors in the ventricular zone of the lateral ventricles and the hypothalamus at E15.5 (Liu et al., 2008). ZEB1 blocks neuronal lineage progression as well as migration of cortical neuroblasts (Wang et al., 2019). Conditional loss of *Zeb1* at E14.5 does not affect cell proliferation or radial glia cell maintenance but causes premature neuronal differentiation (Wang et al., 2019). *Zeb1* overexpression at E14.5 results in reduced neurogenesis, migration defects and subcortical band heterotopia (Wang et al., 2019).

In the adult brain, ZEB1 is important for the self-renewal of adult hippocampal radial glia-like (RGL) cells. *Zeb1* loss in RGL cells results in their precocious differentiation into the neuronal lineage (Gupta et al., 2021). This is accompanied by reduced differentiation into the astroglial lineage, but it remains to be resolved whether this is due to an as-yet unspecified role of ZEB1 in glial fate determination or a natural consequence of the increased neurogenesis. Hence, ZEB1 blocks neuronal lineage progression during embryonal and adult neurogenesis. In adult neural stem/progenitor cells, ZEB1 is associated with activation and proliferation, and loss of *Zeb1* results in depletion of the stem cell pool. Contrastingly, ZEB1 functions in neural progenitors during embryonic neurogenesis appear to be separated in time, with *Zeb1* loss affecting proliferation of progenitors in a constitutive knockout model, but not of later radial glia cells when deleted at E14.5 (Liu et al., 2008; Wang et al., 2019). In both adult and embryonic neurogenesis, *Zeb1* loss is associated with a change in cell division type (symmetric vs. asymmetric) which promotes differentiation of the stem/progenitor cell pool. Interestingly, *Zeb1* loss during embryogenesis promoted asymmetric divisions that prevented expansion of neural progenitors (and therefore caused premature differentiation), whereas in the adult hippocampus *Zeb1* KO causes increased symmetric divisions of neural stem/progenitor cells which are necessary for self-renewal, thus promoting their differentiation (Liu et al., 2019; Gupta et al., 2021).

## Conclusion

Although neurogenic transcription factors are expressed during embryonic and adult neurogenesis their functions often show differences during both processes. These differences include increased spatial confinement and spatial heterogeneity in adult neurogenic niches, different activities in neural progenitor cells at various developmental stages, and/or different effects on downstream progenitor cells. It is important to consider epigenetic modifications, post-translational modifications, and differential expression of interacting partners at different developmental stages to unravel the functions of each neurogenic transcription factor at specific points in time and space. For example, changes in ASCL1 post-translational degradation result in different behavior of adult neural stem cells in juvenile and adult hippocampal neurogenesis (Harris et al., 2021). Integrated analysis of neurogenic transcription factors across development and aging is needed to reveal the specific co-factors contributing to the differential functions in embryonic and adult neurogenesis.

## Data availability statement

No new data was created during this study.

## Author contributions

All authors listed have made a substantial, direct, and intellectual contribution to the work and approved it for publication.

## Funding

NS was the recipient of a Biotechnology and Biological Sciences Research Council (BBSRC) PhD studentship (BB/T008741/1). FS was supported by MRC grant MR/S007709/1. IM-G was supported by funding from the BBSRC (BB/S002359/1). Open access publication fees were covered by the Cardiff University’s Open Access Institutional Fund.

## Conflict of interest

The authors declare that the research was conducted in the absence of any commercial or financial relationships that could be construed as a potential conflict of interest.

## Publisher’s note

All claims expressed in this article are solely those of the authors and do not necessarily represent those of their affiliated organizations, or those of the publisher, the editors and the reviewers. Any product that may be evaluated in this article, or claim that may be made by its manufacturer, is not guaranteed or endorsed by the publisher.
